# The completion of partograms: knowledge, attitudes and practices of midwives in a public health obstetric unit in Bloemfontein, South Africa

**DOI:** 10.11604/pamj.2020.36.301.24880

**Published:** 2020-08-18

**Authors:** Hanneke Brits, Gina Joubert, Fulufhelo Mudzwari, Monica Ramashamole, Moipone Nthimo, Ntšebo Thamae, Mamello Pilenyane, Maphuti Mamabolo

**Affiliations:** 1 Department of Family Medicine, Faculty of Health Sciences, University of the Free State, Bloemfontein, South Africa,; 2 Department of Biostatistics, School of Biomedical Sciences, Faculty of Health Sciences, University of the Free State, Bloemfontein, South Africa,; 3 University of the Free State, Bloemfontein, South Africa

**Keywords:** Partograms, midwives, knowledge, attitude, practice

## Abstract

**Introduction:**

most maternal and 24.3% of infant deaths occur during childbirth. Interventions during childbirth may reduce maternal and neonatal deaths. The Guidelines for maternity care in South Africa (2015) stipulates that all observations during labour should be recorded on a partogram. The objective of this study was to assess the knowledge and attitudes of nursing personnel and to evaluate their practices of completing partograms at National District Hospital, South Africa.

**Methods:**

a two-phase, quantitative, cross-sectional, descriptive study design was used. In phase 1, the knowledge and attitudes of midwives and nurses were evaluated. Midwives and nurses completed anonymous, self-administered questionnaires that assessed their knowledge and attitudes. In Phase 2, partogram practices were measured by assessing completed partograms using a data collection tick sheet.

**Results:**

twelve of the 17 nursing personnel completed the questionnaires. More than 90% of participants answered basic partogram knowledge questions correctly, but only two thirds knew the criteria for obstructive labour and just more than half that for foetal distress. Participants displayed a positive attitude toward the use of partograms. Of the 171 randomly selected vaginal deliveries during the study period, only 57.1% delivered with a completed partogram. Most elements of foetal monitoring and progress of labour scored above 80%, however, for maternal monitoring scored poorly in 26.4% of cases.

**Conclusion:**

although 71.4% of partograms scored more than 75% for completion, the critical components that influence maternal and foetal death, like the identification of foetal distress, maternal wellbeing and progress of labour, were lacking.

## Introduction

Worldwide, 830 women die daily (302 000 yearly) [1], either during childbirth or due to a complication of pregnancy. A further 2.7 million neonatal deaths [2] and 1.3 million intrapartum stillbirths [3] occur yearly. In response to these deaths, the United Nations included maternal and child health in their Millennium Development Goals, and the subsequent Sustainable Development Goals [4]. Maternal and child health has also been identified as an area for intervention by the Department of Health in South Africa. A main area for intervention is the updating, training and implementation of national guidelines [5]. Worldwide, maternal mortality has decreased by 44% since 1990 [1], and neonatal mortality by 57% between 2000 and 2015 [2], which confirms the importance of interventions. Because 42% of maternal [6] and 24.3% of infant deaths [7] occur during childbirth, monitoring labour is probably the most critical activity during which interventions to prevent morbidity and mortality of both the mother and the baby can take place. The *Guidelines for maternity care in South Africa* [5] stipulates that all observations of the mother and foetus during labour should be recorded on a partogram, which is a legal document [8].

Professor RH Philpott introduced the modern partogram, which is a graphical recording of the labour, including maternal and foetal observations that chart the progress of labour. The modern partogram is an improvement on the original partogram, developed by Friedman in 1955, as it includes the alert and action lines to identify abnormal labour [9,10]. If abnormal labour can be identified and addressed at an early stage, complications of prolonged and obstructed labour, such as post-partum haemorrhage, sepsis, birth asphyxia and death, can be reduced. An Indian study found a reduction of 50% in post-partum haemorrhage after the routine introduction of the partogram [11]. Despite the low quality of supporting evidence for the use of partograms during labour, the WHO Guideline Development Group made a strong recommendation in favour of the partogram, specifically in under-resourced settings. It is important that the use of partograms should be accompanied by adherence to standard labour management protocols [12]. Many studies, specifically in under-resourced and third-world countries, report good knowledge and attitudes, but poor practice of partograms [13-15].

The aim of this study was to assess the knowledge and attitudes of midwives and nurses and to evaluate their practices of completing partograms at the obstetric unit of the National District Hospital in Bloemfontein, South Africa. National District Hospital is part of the academic training complex of the University of the Free State and is involved in undergraduate and postgraduate training of medical, allied health and nursing students. During 2018, a total of 1 740 normal vaginal deliveries, 832 caesarean sections and 38 assisted deliveries were performed at the setting. At the same time, a further 239 babies were born before they arrived at the facility. Professional nurses take responsibility for the unit, which offers 24-hour doctor assistance and has theatre availability in the daytime.

## Methods

A two-phase, quantitative, cross-sectional, descriptive study design was used. In phase 1, the knowledge and attitudes of midwives and nurses were evaluated. In phase 2, partogram practices were evaluated.

### Phase 1

**Study population and sample**: the study population and sample consisted of all the advanced midwives and professional nurses working in the maternity unit of National District Hospital at the time of data collection.

**Measurement**: midwives and nurses on duty during the study period completed an anonymous, self-administered questionnaire after they were informed about the study and had read the information leaflet. This was done during day and night duty over a two-week period to include most participants. The researchers developed the questionnaire from the *Guidelines for maternity care in South Africa* [5] manual. Completion of the questionnaire implied consent. The questionnaire assessed: 1) knowledge on the use and benefits of the partogram; 2) attitudes and views on the use of the partogram; and 3) opinions on factors that might influence the effective use of the partogram.

### Phase 2

**Study population and sample**: a systematic random sampling method was used to include every third vaginal delivery over three months (July-September 2018) and to assess their partograms. Names and file numbers were gathered from the delivery register and files were collected from the file store.

**Exclusion criteria**: 1) elective caesarean sections were not included during the random sampling; and 2) files of mothers who had delivered before the partogram was started were excluded after selection.

**Measurement**: partograms were assessed using a data collection tick sheet developed from information in the *Guidelines for maternity care in South Africa* [5] as well as the in-house self-assessment audit tool for partograms in labour wards of the Free State province. The way partograms had been completed was assessed under the following headings: Patient information, Date and time intervals, Foetal information, Progress of labour, Contractions, Maternal condition, Management, and Identification. A number was allocated for each item under each heading: 1) not done = 0, 2) partially done = 1, 3) correctly done = 2, 4) not applicable = 3.

**Data management and analysis**: all data from the questionnaires and the tick sheets were captured into a Microsoft Excel 2016 spreadsheet. Data were checked for correctness and completeness by the researchers and verified by the supervisor. Department of Biostatistics, University of the Free State, analysed the data. A score was calculated under each heading to assess for completeness. A score of ≥ 80% per category was considered good, and <50% as poor. The results were summarised by frequencies and percentages (categorical variables) and medians (numerical variables due to skew distributions).

**Validity**: the validity of the questionnaire and data collection sheets was established from information in the literature, discussion with experts in the field and through pre-testing of the collection tools.

**Ethical considerations**: the project was approved by the Health Sciences Research Ethics Committee of the University of the Free State (HSREC UFS-HSD2018/0417/2602) and the Free State Department of Health. All data were managed confidentially, and no health care professional or patient is identified in any reporting of data.

## Results

A total of four advanced midwives and eight professional nurses completed the questionnaires, while two were on leave and one did not report for work during the data collection period. Two chose not to complete the questionnaire. The response rate was 70.6%.

### Measurement of knowledge and attitudes

**Baseline data**: one-third of respondents were advanced midwives and two-thirds held nursing diplomas. They had worked in a maternity ward for between 2 months and 17 years (median = 3 years), with 41.7% (n=5) working there for at least 9 years.

**Knowledge**: most participants gave the correct answers for the basic knowledge questions e.g. the components of a partogram (91.7%), the duration of latent and active labour (91.7%) and the use of the partogram to identify prolonged labour and foetal distress (83.3%). Between 54.6% and 66.7% answered correctly regarding how often to monitor the mother and foetus, as well as how to identify obstructive labour and foetal distress. The knowledge results are displayed in Table 1.

**Table 1:** percentage of participants who answered the knowledge questions correctly

Question	% correctly answered
Knew that the partogram is a legal document	91.7
Could correctly identify the components on a partogram	91.7
Knew the time intervals for monitoring maternal and foetal wellbeing	66.7
Knew the normal duration of the latent and active phases of labour	91.7
Knew that information on a partogram can be used to identify prolonged labour	83.3
Knew that information on a partogram can be used to identify foetal distress	83.3
Knew that information on a partogram can be used to interpret contractions	75.0
Could correctly identify signs of obstructive labour	66.7
Could correctly identify signs of foetal distress	54.6

**Attitudes**: the participants displayed positive attitudes toward the use of partograms. Two participants (16.7%) stated that the partogram is useful, nine (75.0%) indicated it is very useful, and only one (8.3%) did not find it useful at all. Most (83.3%) stated that partograms make caring for patients easier, and 91.7% indicated that partograms help them to identify possible complications. The factors mentioned that influence the completion of partograms are time constraints and personnel shortages. Although all participants knew that the partogram should be filled in during the labour process, 41.7% (n=5) found it difficult to divide their attention between the patient and the partogram. Shortages of personnel and busy units force participants to fill in partograms after the labour, or they do not complete it at all. One participant indicated that it is important that the partogram contains no mistakes and, therefore, partograms are “corrected” after the delivery. A quarter of participants (n=3) indicated that they had not received any formal training on completing partograms.

**Figure 1:**
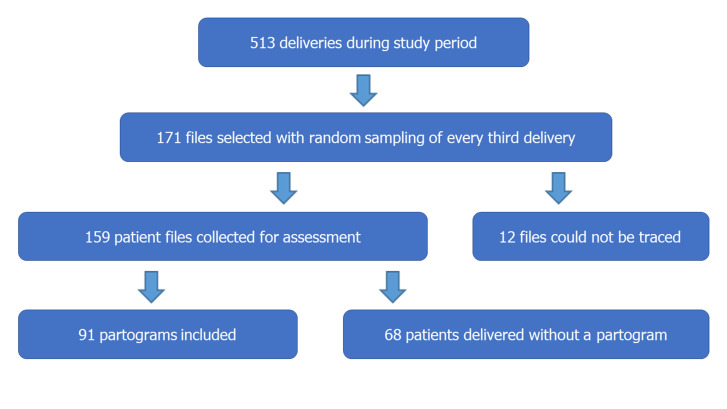
flow diagram of files selected and included in the study

**Measurement of practices**: a total of 91 partograms were assessed. Figure 1 is a flow diagram depicting how files were selected and included. In Phase 1, ten participants (88.3%) indicated that they fill in partograms for every patient in labour. Despite this, only 57.2% (n=91) of the collected files had completed partograms and could be included in the study. Of those, just more than half (56.0%) had more than one entry on the partogram. Patient baseline information was recorded well, except for the duration of labour that was only recorded in 45.1% of cases. Foetal well-being scored above 80%, except for decelerations that scored only 57.1%. Cervical dilatation as an indication of progress of labour was recorded in 96.7% of cases, while the position of the head was only recorded in 26.4%. The number and strength of contractions were recorded on 96.7% of partograms. Table 2 indicates the percentage of partograms filled in correctly for specific parameters under each heading. A score of ≥80% was considered good practice, while a score of < 50% was considered poor practice. Patient baseline information, foetal assessment and assessment of contractions were the categories for which good practice was demonstrated. Regarding the identification of the health care worker that completed the partogram, good practice was demonstrated in only 2.2% of cases (Table 3). The median score for completion of the entire partogram was 81.1% (range 35.1% to 97.3%).

**Table 2:** percentage of each parameter filled in correctly under each heading

Parameter	Percentage (%)
**Patient baseline information**	
Name	100.0
Age	96.7
Gravity	98.9
Parity	97.8
Gestational age	86.8
Spontaneous/Induced labour	89.0
Risk factors	83.5
Time of rupture of membranes	75.8
Pelvis assessment	98.9
Duration of labour on arrival	45.1
**Date and time intervals**	
Date	78.0
Time	98.9
2-hour intervals*	66.7
**Foetal assessment**	
Foetal heart rate	81.3
Decelerations	57.1
Liquor	84.6
Station	93.4
Presenting part	94.5
Caput	94.5
Moulding	94.5
**Progress of labour**	
Position of head	26.4
Head above brim	89.0
Cervical dilatation	96.7
Cervical length	73.6
**Contractions**	
Number of contractions	96.7
Strength of contractions	96.7
**Maternal assessment**	
Blood pressure	89.0
Pulse	82.4
Urine volume	63.7
Urine dipsticks	68.1
Temperature	49.5
**Management**	
Time	60.4
Medication/IV fluids	80.2
Pain relief	83.5
**Identification**	
Time	60.4
Name	12.1
Signature	96.7
Rank	15.4


* Only 51 patients had more than one recording on the partogram, therefore, the time interval could only be assessed for those cases (missing frequency n = 40).

**Table 3:** percentage of partograms demonstrating poor and good practice per category

Partogram category	Poor practice (<50%)	Good practice (≥80%)
Patient baseline information	2.2%	84.6%
Date and intervals	2.2%	61.5%
Foetal assessment	5.5%	79.1%
Progress of labour	8.8%	58.2%
Contractions	2.2%	94.7%
Maternal assessment	26.4%	63.7%
Management	18.7%	58.2%
Identification	30.8%	2.2%

## Discussion

The response rate of more than 70% for the questionnaires, as well as the demographic distribution of the participants, make the data collected representative of that of the nursing personnel at National District Hospital. This information is, however, not necessarily transferable to other settings, as this setting is a district hospital that is part of an academic training complex. More than 90% of participants answered basic knowledge items, for instance, the components on a partogram, correctly, but much lower percentages answered higher-level interpretation items correctly. Only two-thirds knew the criteria for obstructive labour, and just more than half for foetal distress. Taking into consideration that the aim of a partogram is to alert health care workers of foetal distress, maternal abnormalities and poor progress of labour [16], birth attendants should have adequate knowledge to identify these conditions. Other African studies, in Cameroon, Nigeria, Eritrea, Ghana and Ethiopia, found the same lack of knowledge regarding the interpretation of results [15, 17-19]. Training to improve knowledge may seem like a logical step; however, a study performed in Nigeria concluded: “Training improved knowledge, but not the use of the partogram by health care workers” [14].

In this study, the attitudes of the health care workers were positive regarding the completion of partograms, with more than 90% believing that partograms are useful and could help them to identify problems during labour. Although a positive attitude towards the usefulness of partograms was identified, time constraints, lack of personnel and emphasis on the correct filling in of partograms sometimes meant participants did not fill in partograms before after the delivery, or to “correct” mistakes on the partogram. In an Eritrean study, 88.7% of participants had a positive attitude about the filling in of partograms [15], while most other African studies mention a lack of printed partograms, poor knowledge, and staffing issues as reasons for negative attitudes towards the filling in of partograms [17-20]. Although 25% of participants in this study indicated that they had not received any formal training on the completion of partograms, it was not mentioned as a reason for failing to fill in partograms. Despite almost 90% of participants indicating that they fill in a partogram for every patient who delivers, less than 60% of the patients delivered with a completed partogram. This finding must be taken into consideration when these results are interpreted. A reason for the lack of partograms may be that many patients present very late in labour (a deduction supported by a large number of babies born before arrival at the hospital), and that just more than half of the patients had more than one recording on the partogram before they delivered. The reported completion rate for partograms was 79.4% in a South African study [21], but much lower for the rest of Africa, with figures of around 35% [18-20]. These low rates of partogram utilisation may be a reason why the Cochrane review could not find high-quality evidence of the impact of partogram use on maternal and infant outcomes in resource-limited areas [16]. Another reason may be that practices were reported on and not measured. Patient baseline information scored well, with all patients identified on the partograms. To diagnose prolonged labour, it is important to know the duration of labour before the patient arrived at the hospital. This parameter was, however, not recorded for more than half of the patients.

All components of foetal assessment scored above 80%, except decelerations, which is the main indicator of foetal distress. Two recent African studies, one in Zambia [22] and one in Malawi [23], reports on practices relating to partogram completion. These studies did not report on decelerations, but only on foetal heart rate, which had 82.6% and 85.1% completion rates for the two studies respectively, compared to the 81.3% in this study. Progress of labour was assessed by cervical dilatation and the descent of the foetal head, and in our and the two African studies, it scored above 80%. Maternal monitoring, however, scored less than 80% in all three studies. In a quarter of cases, the monitoring of the mother scored less than 50%. Taking into consideration that almost half of maternal deaths occur during labour [7], better maternal monitoring should be addressed urgently. According to the Health Professions Council of South Africa, a partogram is a record of care and, therefore, a legal document that must contain the personal particulars of the patient, a history, time and date of consultation, assessment findings and a management plan. All legal documents must be dated and signed next to the initial and surname in block letters [8]. When assessing partograms as legal documents, all had patient identification details, all contained some history and assessment, three quarters had a date and just more than half had a time recorded with interventions, however, the health care professional could only be identified by a printed name in one in eight cases.

**Limitations**: due to the setting, these results may not be representative of other settings in South Africa. Despite an above 70% completion rate of questionnaires, the numbers of cases and participants are small. The large number of patients that delivered without a partogram or with only one recording on the partogram may have skewed the results; however, it highlighted the real-life situation of late presentation at labour wards, which is accompanied by its complications. The time of partogram completion (day versus night or weekday versus weekend) was not assessed. The difference between reported and measured practice must be taken into consideration when results are interpreted and compared.

## Conclusion

Although more than 70% of the partograms scored more than 75% for completion, it was the critical components that influence maternal and foetal death, such as the identification of foetal distress, maternal wellbeing, and progress of labour, that were lacking. Regular training and monitoring of partogram completion may be a first step to improving the practice of partogram use.

### What is known about this topic

- The correct completion of partograms can save mothers and babies during childbirth;
- A partogram should be used to monitor each delivery.

### What this study adds

- Partograms are not used optimally and therefore important danger signs are missed;
- Many deliveries take place without the filling of a partogram.
